# A Flexible Chemosensor Based on Colorimetric and Fluorescent Dual Modes for Rapid and Sensitive Detection of Hypochlorite Anion

**DOI:** 10.3390/s21238082

**Published:** 2021-12-03

**Authors:** Qin Wu, Tao Tao, Yunxia Zhao, Wei Huang

**Affiliations:** 1 Institute of Advanced Materials and Flexible Electronics (IAMFE), School of Chemistry and Materials Science, Nanjing University of Information Science & Technology (NUIST), Nanjing 210044, China; 20191213038@nuist.edu.cn (Q.W.); nlgzyx@nuist.edu.cn (Y.Z.); 2 School of Chemistry and Chemical Engineering, Nanjing University, Nanjing 210093, China; whuang@nju.edu.cn

**Keywords:** aggregation-induced emission, chemosensor, tetraphenylethylene, hypochlorite, test paper

## Abstract

A flexible chemosensor has been developed based on colorimetric and fluorescent dual modes using tetraphenylethylene-centered tetraaniline (**TPE4A**) for rapid and sensitive detection of hypochlorite anion. The fluorescent probe **TPE4A** exhibits a unique aggregation-induced emission (AIE) character which is proved by a blue shift of the fluorescent peak from 544 to 474 nm with the water equivalents increasing. With the addition of hypochlorite in solution, the absorbance of the probe changes and the responding fluorescence color can be observed to change from light green to purple. The detection limit of hypochlorite is 1.80 × 10^−4^ M in solution, and the visual detection limit is 1.27 µg/cm^2^ with the naked eye for the flexible paper-based chemosensor. The proposed flexible chemosensors show a good selectivity and sensitivity which has great potential for effective detection of hypochlorite anions without any spectroscopic instrumentation.

## 1. Introduction

Hypochlorite anion (ClO^−^) plays an essential role in biological organisms and environmental monitors, which not only is produced by H_2_O_2_ and chloride ions in activated neutrophils [1,2,3,4,5] but is also a kind of disinfector which can kill the coronavirus [6,7,8]. However, excessive hypochlorite could lead to diseases and even cancer including cardiovascular diseases, neuron degeneration, and arthritis [9,10]. Therefore, quantitative detection of hypochlorite with highly sensitive and selective methods becomes more and more crucial.

Many various analytical methods are available for the detection of hypochlorite (for example, colorimetric and fluorescent chemosensors) [11,12]. Recent reviews have summarized the advances of hypochlorite probes [13,14]. There are some successful designs for quantitative detection of hypochlorite based on the principle of colorimetric, chromatographic, electrochemical, and luminescent methods. Specifically, the fluorescent probe is a promising method with advantages such as low cytotoxicity, high selectivity, and fast response time for ClO^−^ detection. Moreover, understanding visual detection is an unceasingly thorough process for rapid and sensitive detection of hypochlorite anions [15,16,17,18,19,20,21,22,23,24].

**Scheme 1:**
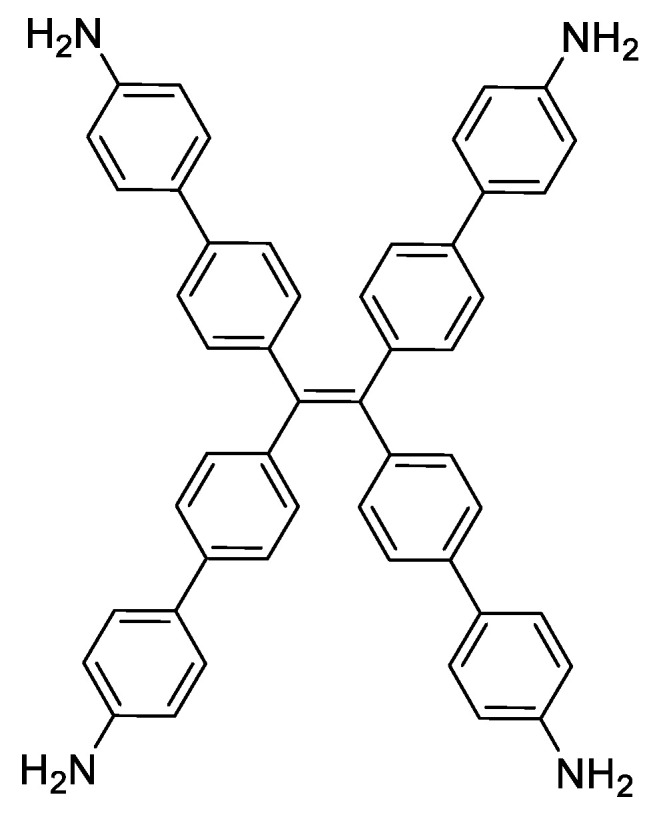
The chemical structure of tetraphenylethylene-functionalized molecule **TPE4A**.

Recently, dual modes [25,26,27] bioanalysis has become a popular research area, wherein researchers are studying scrambly as well. When the environment changes in the direction of complexity, single-switch optical detection may no longer meet the reliability of data and the diversity of application scenarios. Therefore, it is very important to construct a dual-mode optical detection method. The optical detection based on colorimetric and fluorescent dual modes could not only improve the accuracy of results but also lead to higher efficiency. In recent years, flexible thin-film devices [28,29] have been developed vigorously, especially in material, chemical, biological, physical fields, due to their unique advantages such as their low-cost, porosity, ready availability, mechanical flexibility, etc. Therefore, the design of paper-based chemosensors for simple and rapid detection is of great significance. In this work, we present a commercially available material **TPE4A** for fabricating a flexible paper-based chemosensor based on colorimetric and fluorescent dual modes in Scheme 1.

## 2. Results and Discussion

### 2.1. NMR Spectra

A commercially available material **TPE4A**, namely 4′,4‴,4′′′′′,4′′′′′′′-(ethene-1,1,2,2-tetrayl)-tetrakis(([1,1′-biphenyl]-4-amine)) [30,31], has been purchased where the tetraphenylethylene (TPE) group shows an aggregation-induced emission (AIE) character [32,33]. The chemical structure of **TPE4A** has double checked by NMR spectra in Figure S1. ^1^H NMR (400 MHz, *d*^6^-DMSO): *δ* 7.37 (d, *J* = 8.3 Hz, 8H, TPE), 7.34 (d, *J* = 8.4 Hz, 8H, Phenyl), 7.03 (d, *J* = 8.3 Hz, 8H, TPE), 6.59 (d, *J* = 8.4 Hz, 8H, Phenyl), 5.22 (s, 8H, NH_2_).

### 2.2. Aggregation-Induced Fluorescent Behavior

**Figure 1:**
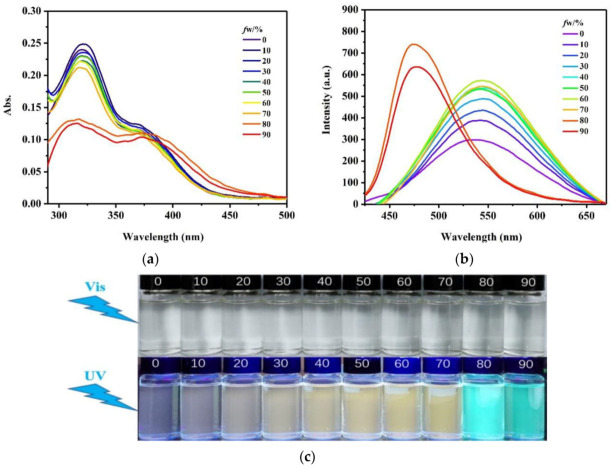
UV–Vis absorption (**a**) and fluorescent emission (**b**) spectra and their visual photograph (**c**) for compound **TPE4A** with different *f*_w_ in their THF/water solutions with the same concentration of 10 µM.

With the enhancement of volume fraction of water phase (*f*_w_), UV–Vis and fluorescent spectra have been shown for compound **TPE4A** in Figure 1. A step-by-step fluorescent turn-on is observed from *f*_w_ = 0 to 70%, due to the controlled rotational motion in the molecule. Interestingly, a remarkable color change occurs from orange when *f*_w_ = 70% to green when *f*_w_ = 80% mainly due to the formation of aggregates. Simultaneously, the maximum emission wavelength *λ*_max_ is from 544 nm when *f*_w_ = 70% to 474 nm when *f*_w_ = 80%. Absorption and emission spectra show a clear AIE character for compound **TPE4A** as expected. The dominant luminescence is from the emissive solution to solid fluorescence. The same phenomenon is observed in the different concentration measurements. According to restriction of intramolecular vibrations (RIV) and restriction of intramolecular rotations (RIR), the possess of twisted intramolecular charge transfer (TICT) also is blocked, resulting in decreasing red-shift and increasing blue-shift.

### 2.3. Titration Experiment of Probe TPE4A

**Figure 2:**
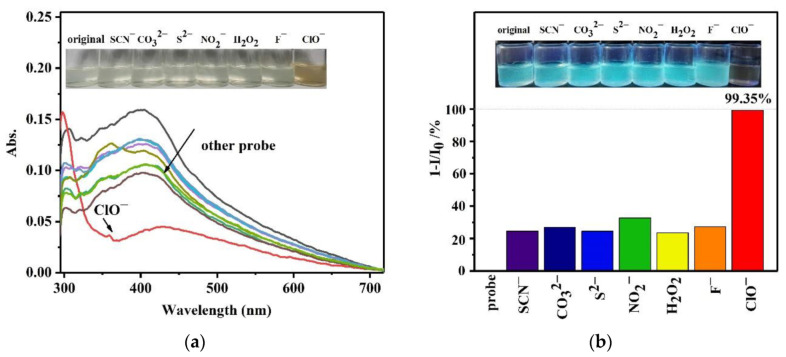
UV–Vis absorption (**a**) and fluorescent quenching percentages at 480 nm (**b**) of probe **TPE4A** (40 µM) in mixed solution (*f*_w_ = 80%) upon exposure to other analytes for 60 s. Inset: a colorimetric and fluorescent change photograph for ClO^−^ and other analytes (SCN^−^, CO_3_^2−^, S^2−^, NO_2_^−^, H_2_O_2_, and F^−^).

Compared to other chemical anions, molecule **TPE4A** has been designed as a highly selective fluorescent probe for ClO^−^ anion in Figure 2. After adding 1.00 mM of analytes, such as SCN^−^, CO_3_^2−^, S^2−^, NO_2_^−^, H_2_O_2_, F^−^, and ClO^−^, UV–Vis absorption, and fluorescent intensities of probe **TPE4A** (40 µM) have been shown in mixed solution (*f*_w_ = 80%). The absorption peak of **TPE4A** is from 398 nm to 429 nm (also from 3.12 eV to 2.89 eV) in the presence of ClO^−^, which is consistent with the results of calculated energy gaps.

**Figure 3:**
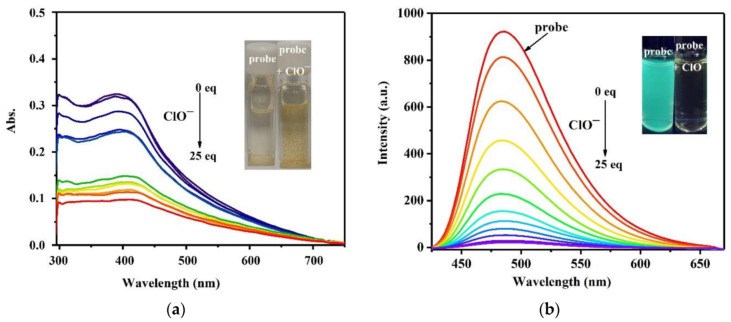
Absorption (**a**) and emission (**b**) titration spectra of the probe **TPE4A** (40 µM) in the presence of various concentrations of ClO^−^ from 0 to 25 equivalent in mixed solution (*f*_w_ = 80%). Inset: the contrastive pictures before and after the addition of ClO^−^.

More importantly, a fluorescent quenching occurs from light green to dark, while the emissive color of this probe remains unchanged after the adding of other chemical species. Quantitative titration experiment shows a clear fluorescent turn-off in the presence of different concentrations of hypochlorite anion from 0 to 25 equivalent in mixed solution (*f*_w_ = 80%) with 100% of quenching percentage in Figure 3. It is noted that the intensity of **TPE4A** at 480 nm does not significantly change under H_2_O_2_ or ClO_4_^−^ oxidants, since this compound avoids being oxidized in this case.

### 2.4. Paper-Based Sensor

**Figure 4:**
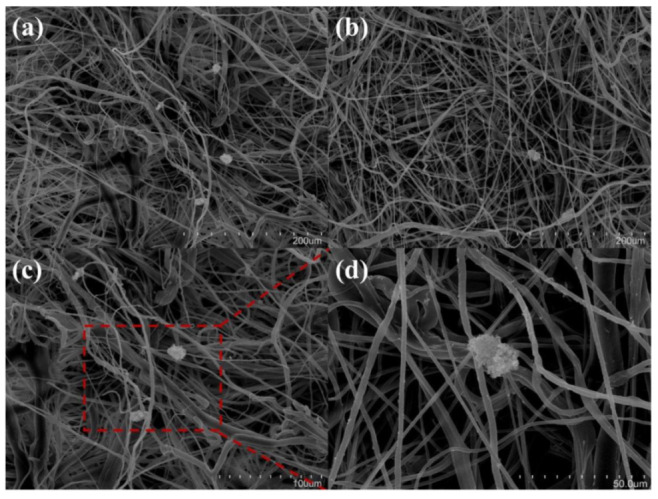
SEM images in different sizes of paper-based sensor **TPE4A**: 200 µm (**a**,**b**), 100 µm (**c**), and 50 µm (**d**).

**Figure 5:**
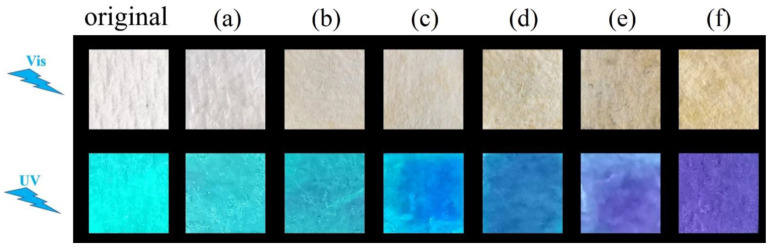
The naked-eye recognition matrix diagram of paper-based sensor **TPE4A** for different concentrations of ClO^−^ under visible and UV light. Original 0 M, (**a**) 5.0 × 10^−4^ M, (**b**) 1.0 × 10^−3^ M, (**c**) 5.0 × 10^−3^ M, (**d**) 1.0 × 10^−2^ M, (**e**) 5.0 × 10^−2^ M, (**f**) 1.0 × 10^−1^ M.

To effective detection of ClO^−^ in the state of aggregation, we prepared a test paper as the paper-based sensor. Then, we dipped the test paper into a solution containing chromophore test paper. As shown in Figure 4, the SEM diagram indicates that **TPE4A** can be attached to paper-based fibers. The diameter of particulate matter is approximately 100 µm, while the diameter of melt-blown fibers is 20~50 µm, showing a size matching on a micron scale. We carried out the naked-eye recognition matrix of paper-based sensor **TPE4A** for different concentrations of ClO^−^ under visible and UV light in Figure 5. The fluorescence of the paper-based sensor is quenched gradually with the increase of the concentration of ClO^−^. At the same time, the color of the sensor changes from white to yellow under visible light, while from green to purple with the light of the UV lamp at 365 nm. Quantitative analysis demonstrates that the detection limit of hypochlorite is 1.80 × 10^−4^ M in solution based on the IUPAC definition 139 (*C*_DL_ = 3 Sb m^−^^1^) [34], and the visual detection limit is 1.27 µg/cm^2^ with the naked eye. Therefore, tetraphenylethylene-centered tetraaniline materials have a rapid and highly sensitive character for more convenient and visual detecting hypochlorite in a few seconds.

### 2.5. Possible Mechanism of Probe TPE4A

**Scheme 2:**
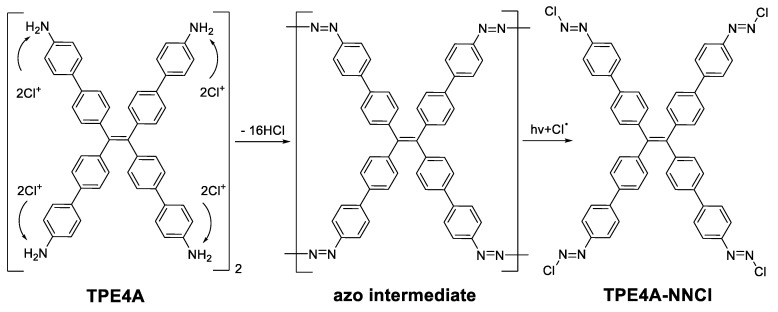
Possible mechanism of chemosensor **TPE4A** for ClO^−^.

According to the references [16,35,36], as well as experimental and theoretical calculations [37], we have investigated the possible mechanism of **TPE4A** in Scheme 2. First of all, the tetraaniline structure may be oxidized to the azo counterpart under an appropriate oxidant in this case. It is noted that perchloride is a stronger oxidant meant it could oxidize the aniline group to form the azo intermediate. However, the solubility of perchloride is limited in *f*_w_ = 80%. The mechanism of fluorescent turn-off quenching is a reaction from strong chromophore **TPE4A** to weak chromophore **TPE4A-NNCl**, which could be speculated that owing to the oxidation of **TPE4A**. Furthermore, the C–N bond could be easy to cleavage in the azo intermediate and further to form a radical which could combine with chlorine radicals to form the compound **TPE4A-NNCl**. The energy gap is 3.44 eV for **TPE4A**, while the energy gap is 3.17 eV for **TPE4A-NNCl** (see Supporting Information).

In addition, we carried out the ESI-MS analysis to explore the complete reaction mixture of the probe with ClO^−^ in Figure S3. ESI-MS analysis that a peak at *m*/*z* = 837.1954 corresponding to [**TPE4A-NNCl**-2Cl+Na]^+^ (calcd for [C_50_H_32_Cl_2_N_8_Na]^+^: 837.2019) is clearly observed with other identical isotopic peaks. This promoted a possible method to design fluorescent probes for hypochlorite with the inorganic/organic composites containing amino groups.

## 3. Conclusions

In summary, we have designed and developed a flexible paper-based material for hypochlorite detection. The quantitative analysis demonstrates that with the enhancement of hypochlorite equivalents, the UV–Vis peaks have been decreasing from 398 nm to 429 nm, while the fluorescent peak at 480 nm has been decreasing more remarkably. Furthermore, the detection limit of hypochlorite is 1.80 × 10^−4^ M in solution, and the visual detection limit is 1.27 µg/cm^2^ with the naked eye. The mechanism of fluorescent turn-off quenching is a reaction from strong chromophore **TPE4A** to weak chromophore **TPE4A-NNCl**, which has been confirmed by experimental and theoretical results, as well as support from references. This study can provide a flexible paper-based portable chemosensor based on colorimetric and fluorescent dual modes for hypochlorite before conventional chemical synthesis.

## Data Availability

Not applicable.

## Supplementary Materials

The following are available online at https://www.mdpi.com/article/10.3390/s21238082/s1. Figure S1: ^1^H NMR of **TPE4A**; Figure S2: The selectivity of **TPE4A** for ClO^−^, SCN^−^, CO_3_^2^^−^, S^2^^−^, NO_2_^−^, H_2_O_2_, F^−^ and ClO_4_^−^; Figure S3: Fitting plot for compound **1** with the addition of different contentions of **TPE4A** in THF solution to calculate the limit of detection; Figure S4: The experimental and theoretical ESI-MS of the product obtained by mixing probe NaOCl; Table S1: The HOMOs and LUMOs of compounds **TPE4A** and **TPE4A-NNCl**.
