# Supplementary material for: A Flexible Chemosensor Based on Colorimetric and Fluorescent Dual Modes for Rapid and Sensitive Detection of Hypochlorite Anion
Source: Sensors (Basel). 2021 Dec 3;21(23):8082. doi: 10.3390/s21238082 (PMC8659631; doi:10.3390/s21238082)
Supplement: Supplementary file 1 [file sensors-21-08082-s001.zip › sensors-1486708-supplementary.pdf]

Supplementary Materials:

A Flexible Chemosensor Based on Colorimetric and Fluorescent Dual Modes for Rapid and Sensitive Detection of Hypochlorite Anion

Qin Wu [1], Tao Tao [1,2,*], Yunxia Zhao [1] and Wei Huang [2]

[1] Institute of Advanced Materials and Flexible Electronics (IAMFE), School of Chemistry and Materials
Science, Nanjing University of Information Science & Technology (NUIST), Nanjing 210044, China; 20191213038@nuist.edu.cn (Q.W.); nlgzyx@nuist.edu.cn (Y.Z.)

[2] School of Chemistry and Chemical Engineering, Nanjing University, Nanjing 210093, China; whuang@nju.edu.cn

* Correspondence: taotao@nuist.edu.cn

Experimental Details

1. aggregation-induced emission (AIE) experiment

Herein, the 4',4''',4''''',4'''''''-(ethene-1,1,2,2-tetrayl)tetrakis(([1,1'-biphenyl]-4-amine)) (**TPE4A**) was dissolved in tetrahydrofuran (THF) into 400 μM solution (mother liquor). Next, take 10 glass bottles of 10 ml numbered 0-90 and add 1 ml of the above solution to each bottle, and then add deionized water according to the ratio of 0-90% of water content, and finally use tetrahydrofuran to form 10 ml mixed solution. Finally, the data analysis via UV test and fluorescence test were indicated that the moisture content reaches 80%, the fluorescence intensity is maximum.

2. Titration experiment with ClO^-

Prepared 5 mM concentration of hypochlorite solution, 2.5 mL fluorescent probe with a moisture content of 80% was added to a clean colorimetric dish. Add 0-25 equivalent of the above hypochlorite solution to it gradually. The data analysis via UV test and fluorescence test.

3. Selective experimental testing of TPE4A

The mixed solution of the fluorescent probe (40 μM, THF/water = 1:4) got ready first. Afterward, prepared the analytes with a concentration of 1000μM (ClO^-、SCN^-、CO_3^{2-}、S^{2-}、NO^{2-}、H_2O_2、F^-), Then carried the fluorescence test by fluorescence testing device.

4. Preparation of experimental details and testing of paper-based sensor

We used a melt-blown fiber test paper material, cut into a 1 cm × 1 cm square lattice, immersed in a concentration of 40 μM TPE4A solution for 24 h, leaving the fiber paper-based material in full contact with the **TPE4A** solution. Next, the Paper-based sensor was removed to dry for standby. Preparation of serial concentrations of ClO^- (5.0×10^{-4} M, 1.0×10^{-3} M, 5.0×10^{-3} M, 1.0×10^{-2} M, 5.0×10^{-2} M, 1.0×10^{-1} M). Then a drop of different concentrations of ClO^- solution was dropped to the paper-based sensor, and fluorescence intensity changes were observed and photographed under a 365 UV lamp and recorded. After the sensor is dried, the color change of the sensor was visually observed and the photo was recorded.

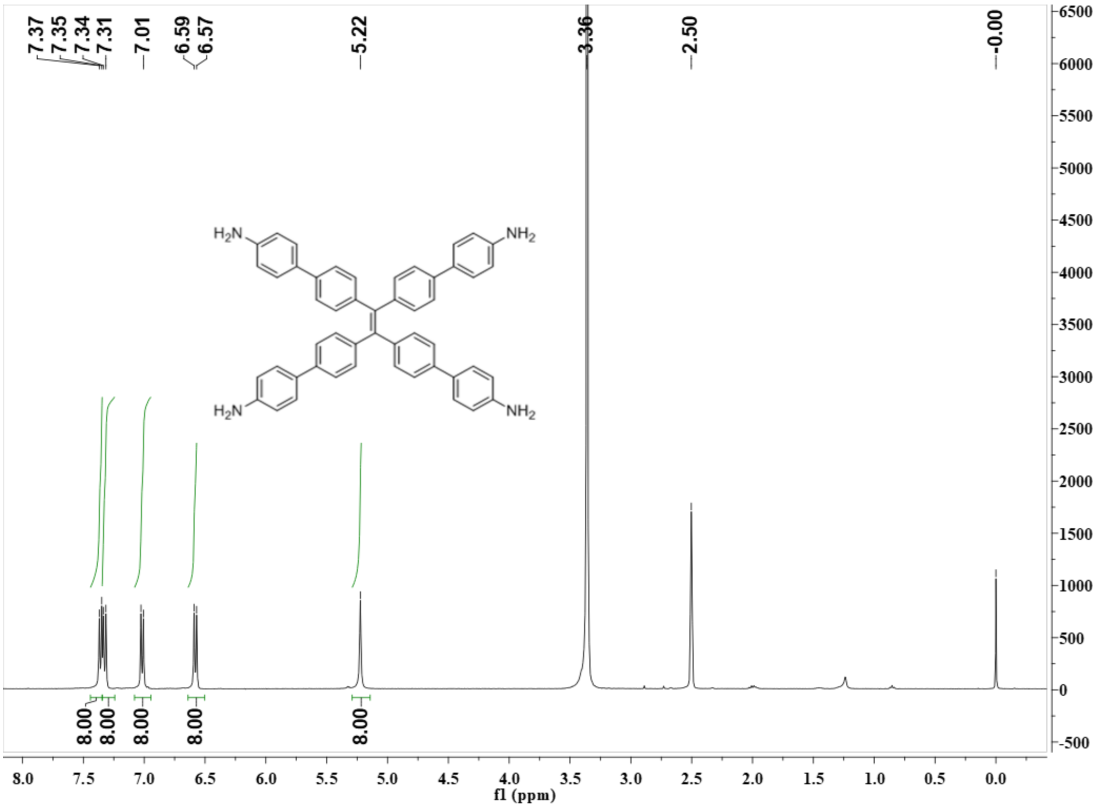

Figure S1. ^{1}H NMR of **TPE4A** in d^6-DMSO

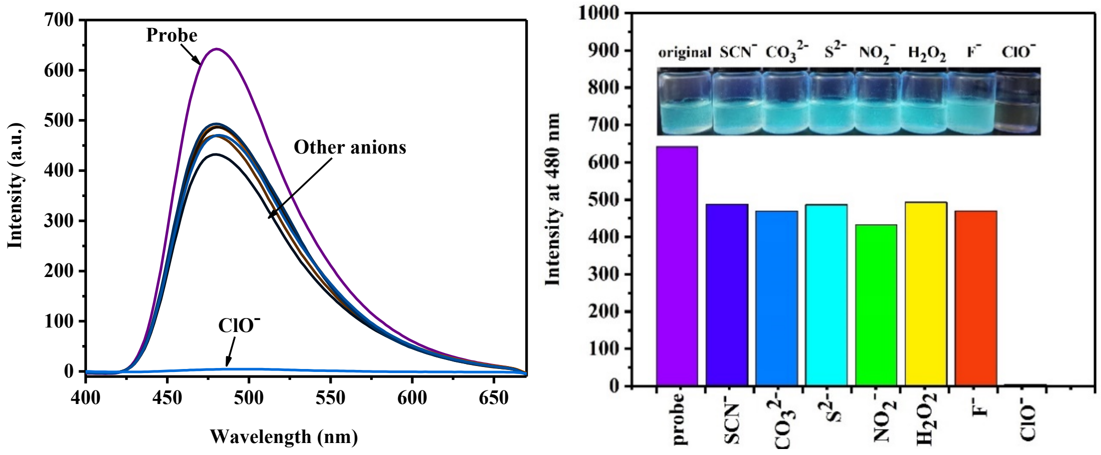

a b

Figure S2. Fluorescent emission (**a**) and intensities at 480 nm (**b**) of probe **TPE4A** (40 μM) in mixed solution (f_w = 80%) upon exposure to other analytes (SCN^-, CO_3^{2-}, S^{2-}, NO_2^-, H_2O_2 and F^-). Inset: selective fluorescent change photograph for ClO^- and other analytes.

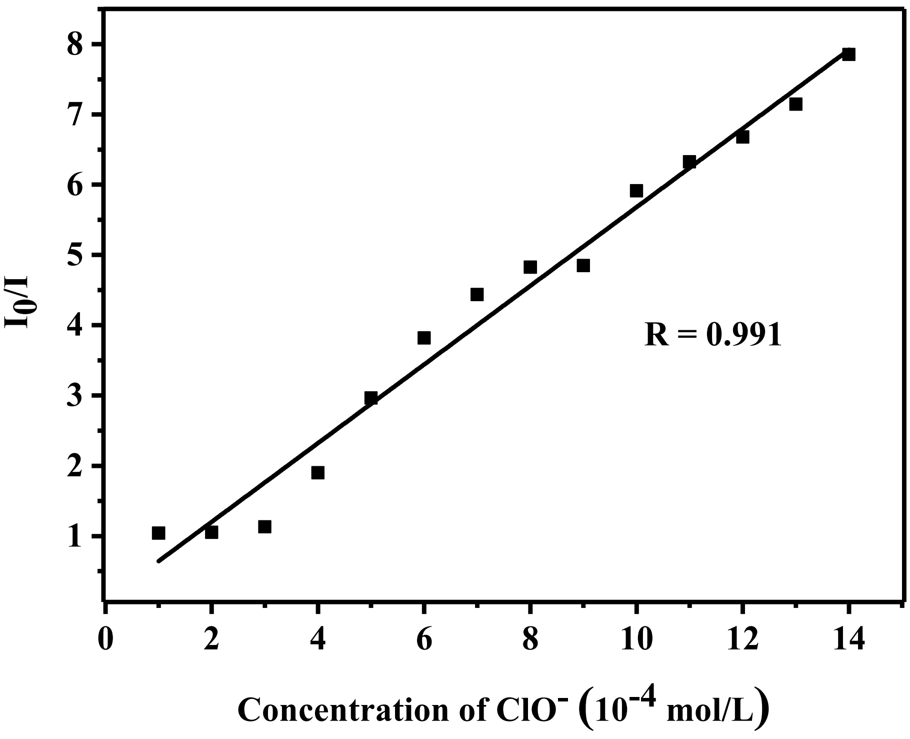

Figure S3. Fitting plot for compound **1** with the addition of different contentions of **TPE4A** in THF solution to calculate the limit of detection.

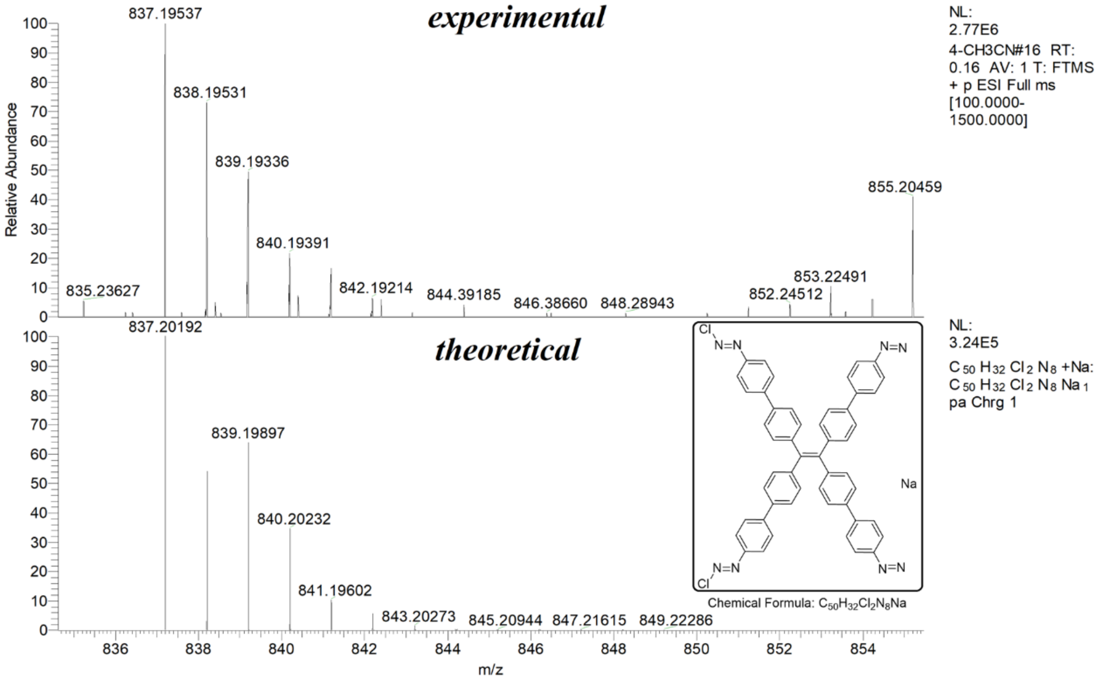

Figure S4. The experimental and theoretical ESI-MS of the product obtained by mixing probe NaOCl

Table S1. The HOMOs and LUMOs of compounds **TPE4A** and **TPE4A-NNCl**.

compound	HOMOs	LUMOs
**TPE4A**	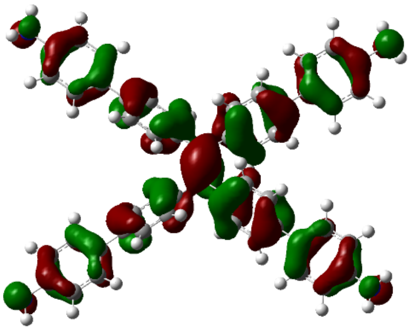 −4.62 eV	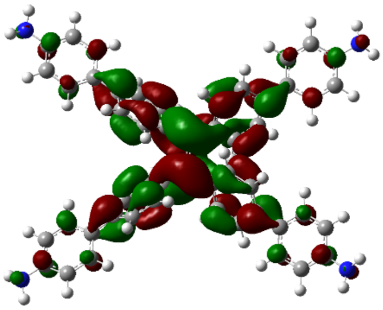 −1.18 eV
**TPE4A-NNCl**	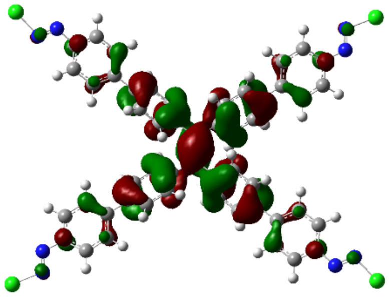 −5.98 eV	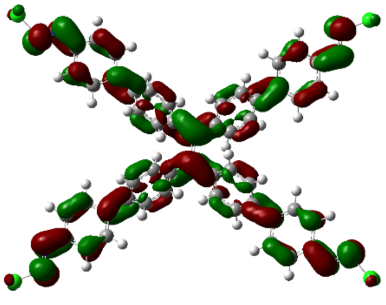 −2.81 eV
